# United Confederate Veterans

**Published:** 1894-04

**Authors:** 


UNITED CONFEDERATE VETERANS.—Surgeon General U. C. V., Joseph Jones, M. D., LL. D., has permanently organized the Medical Department of this organization by the division of the Southern States into three general departments, and assaigning to each department a medical director, who will be commissioned by the General Commanding, Gen. John B. Gordon, and given rank as Brigadier General. The Department of the Atlantic is assigned to Medical Director Hunter McGuire, M. D., Richmond, Va. Department of the Gulf, to Medical Director D. W. Yandell, M. D., Louisville, and the Trans. Mississippi Department to Medical Director J. M. Keller, M. D., Hot Springs. Each State is also made a department, and to each is assigned both a medical director and a medical inspector or two medical inspectors. The medical directors and medical inspectors of States will be commissioned, and will take rank respectively as Colonel and Lt. Colonel.

We note in addition to the above, only the appointments for Texas, to-wit:

S. H. Stout, A. M., M. D., LL. D., Dallas, Texas, late Surgeon C. S. A., and Medical Director of Hospitals, Army Tennessee, Medical Director.

F. E. Daniel, M. D., Austin, Texas, late Surgeon C. S. A., and Judge Advocate Department of Tennessee, Medical Inspector.

Elias J. Beall, M. D., Fort Worth, late Chief Surgeon, Walker's Division, C. S. A., Medical Inspector.

The Surgeon General says: " * * * The fact that the Confederate cause was lost does not the less entitle the Confederate veterans in any and every corps, medical as well as infantry, cavalry and artillery to the gratitude and lasting expression of honorable consideration of their fellow citizens."

At the same time we doubt the wisdom of keeping up such organization.

